# Whey Protein Components - Lactalbumin and Lactoferrin - Improve Energy Balance and Metabolism

**DOI:** 10.1038/s41598-017-09781-2

**Published:** 2017-08-30

**Authors:** Rizaldy C. Zapata, Arashdeep Singh, Adel Pezeshki, Traj Nibber, Prasanth K. Chelikani

**Affiliations:** 1 Department of Production Animal Health, Faculty of Veterinary Medicine, University of Calgary, 3330 Hospital Drive NW, Calgary, Alberta T2N 4N1 Canada; 2 Department of Animal Science, Oklahoma State University, Stillwater, Oklahoma 74078 USA; 3 Advanced Orthomolecular Research, 3900 12 St NE, Calgary, Alberta T2E 6X8 Canada; 4 Gastrointestinal Research Group, Snyder Institute for Chronic Diseases, University of Calgary, 3330 Hospital Drive NW, Calgary, Alberta T2N 4N1 Canada

## Abstract

Whey protein promotes weight loss and improves diabetic control, however, less is known of its bioactive components that produce such benefits. We compared the effects of normal protein (control) diet with high protein diets containing whey, or its fractions lactalbumin and lactoferrin, on energy balance and metabolism. Diet-induced obese rats were randomized to isocaloric diets: Control, Whey, Lactalbumin, Lactoferrin, or pair-fed to lactoferrin. Whey and lactalbumin produced transient hypophagia, whereas lactoferrin caused prolonged hypophagia; the hypophagia was likely due to decreased preference. Lactalbumin decreased weight and fat gain. Notably, lactoferrin produced sustained weight and fat loss, and attenuated the reduction in energy expenditure associated with calorie restriction. Lactalbumin and lactoferrin decreased plasma leptin and insulin, and lactalbumin increased peptide YY. Whey, lactalbumin and lactoferrin improved glucose clearance partly through differential upregulation of glucoregulatory transcripts in the liver and skeletal muscle. Interestingly, lactalbumin and lactoferrin decreased hepatic lipidosis partly through downregulation of lipogenic and/or upregulation of β-oxidation transcripts, and differentially modulated cecal bacterial populations. Our findings demonstrate that protein quantity and quality are important for improving energy balance. Dietary lactalbumin and lactoferrin improved energy balance and metabolism, and decreased adiposity, with the effects of lactoferrin being partly independent of caloric intake.

## Introduction

The guidelines for the management of obesity by the American College of Cardiology, American Heart Association and The Obesity Society recommend inclusion of dietary protein at a minimum 25% of total calories, together with caloric restriction, to promote weight loss in obese adults^1^. Among protein sources, dairy proteins^2, 3^, whey protein in particular^4–8^, decrease body weight and fat, increase lean mass and improve glycemic control in humans. We previously demonstrated that whey is more effective than casein in decreasing food intake, weight and fat gain, and in improving glucose tolerance in rat models of human obesity^9^, hypertension and stroke^10^. Others reported that whey promotes muscle glycogenesis^11^ and decreases hepatic lipidosis^12, 13^ by modulating key regulatory steps of glucose and lipid metabolism in rodent models. However, less is known of the bioactive fractions of whey that improve energy balance and metabolism.

Whey protein is composed of several bioactive fractions including glycomacropeptide, β-lactoglobulin, α-lactalbumin and lactoferrin, with multiple health benefits against cancer, infection and inflammation^14^. Dietary lactalbumin was found to suppress hunger in humans^15, 16^, decrease weight gain and adiposity in rats^12^, mice^17, 18^ and minipigs^19^, and improve glucose tolerance in diabetic rats^20^ and minipigs^19^. Further, lactalbumin and milk protein comparably decrease weight and fat mass in calorie restricted human subjects^21^. Dietary supplementation with lactoferrin has been reported to modulate gut microbiota, decrease weight gain, reduce hepatic lipidosis, and improve glucose tolerance in mice^22, 23^, and produce greater weight and fat loss in calorie restricted mice^17, 24^. Others reported that lactoferrin decreases hepatic lipid content and mesenteric fat without altering food intake, weight gain and body composition in mice^23, 25, 26^. Similarly, in humans, lactoferrin supplementation for 8 weeks^27^, but not 4 weeks^28^, has been shown to decrease visceral adiposity in overweight subjects without altering caloric intake. The reduction in caloric intake with whey-based diets is associated with increased circulating concentrations of satiety hormones of the lower gut including glucagon-like peptide-1 (GLP-1) and peptide YY (PYY) in humans^29–31^, and GLP-1 in rodents^9, 32^. Despite producing hypophagia, lactalbumin does not alter GLP-1 in humans^16, 21, 33^. However, it is unknown whether whey fractions differentially modulate the secretion of these and other satiety hormones. Although, lactalbumin was found to transiently increase energy expenditure in exercising rats^34^, such thermogenic effects are not observed in calorie restricted humans^21^. Thus, despite some evidence for the effects of lactalbumin and lactoferrin on body weight, the relative efficacies of these whey fractions in modulating food intake, energy expenditure and gut hormone secretion, and whether these effects are independent of caloric intake, remain largely unknown.

Our overall objective was to determine the effects of protein quantity and quality on energy balance and metabolism in obese rats. To assess the effects of protein quantity, we compared a normal protein diet with high protein diets containing whey protein components on energy balance and metabolism. To investigate the effects of protein quality, we compared the effects of high protein diets enriched with whey protein isolate, α-lactalbumin or lactoferrin, on food intake, energy expenditure, body composition, glycemic control, and plasma anorexigenic hormones in diet-induced obese rats, and assessed whether such effects were independent of caloric intake. Because we previously noted decreased preference for whey based diets^9, 10^, here, we performed conditioned diet preference test to assess whether the hypophagic effect of test diets was due to altered diet preference. We also determined the transcript and/or protein abundance of regulatory molecules of glucose and lipid metabolism in the liver and skeletal muscle, and quantified select cecal bacterial populations, to gain insights into the mechanisms of metabolic improvements by the whey fractions.

## Results

### Lactoferrin was more effective than whey and lactalbumin in inducing hypophagia

**Figure 1:**
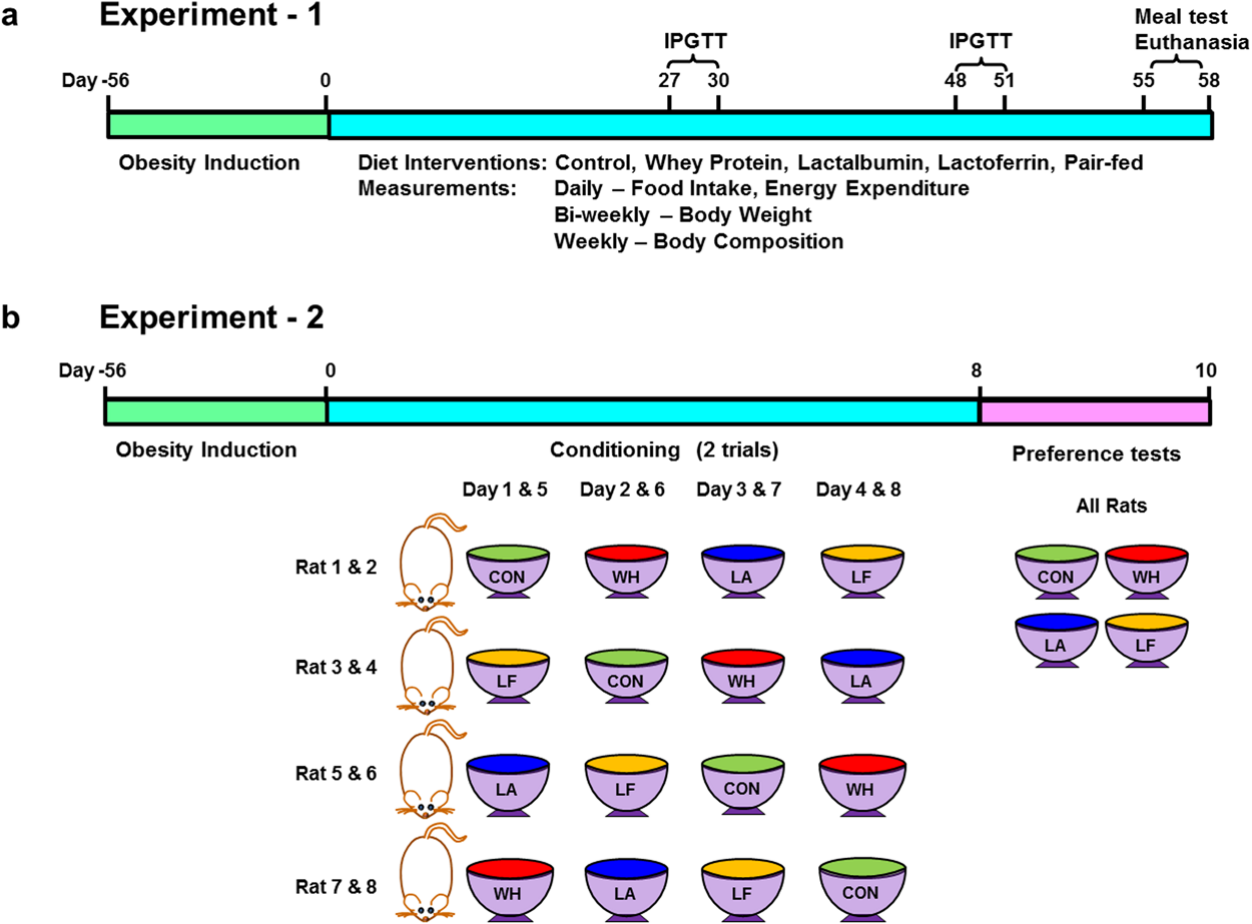
Experimental Timelines. (**a**) *Experiment-1*: Obesity was induced with a high fat diet in male obese prone (OP-CD) rats for ~8 weeks. Next, obese rats (536 ± 7 g, n = 8/group) were randomized to receive one of five isocaloric high fat diets containing either normal protein (control), or high protein diets enriched with whey protein isolate, α-lactalbumin, lactoferrin, or pair-fed to lactoferrin with whey, for 56 days. Daily measurements included food intake and energy expenditure in the Oxymax/CLAMS® system, biweekly body weight, and weekly body composition by Minispec LF-110®. Intraperitoneal glucose tolerance tests (IPGTT) were conducted on days 27–30 and 48–51. Meal tolerance test was performed on days 55–58, followed by euthanasia and tissue sampling for analyses. (**b**) *Experiment-2*: Diet preference was studied in a separate cohort of naïve obese OP-CD rats (413 ± 6 g; n = 8). During an 8-day conditioning, on alternate days, rats had 6-h access to one of the treatment diets flavored with KoolAid®. Following two conditioning periods, preference tests were conducted for  two days with rats having simultaneous access to all four diets.

**Figure 2:**
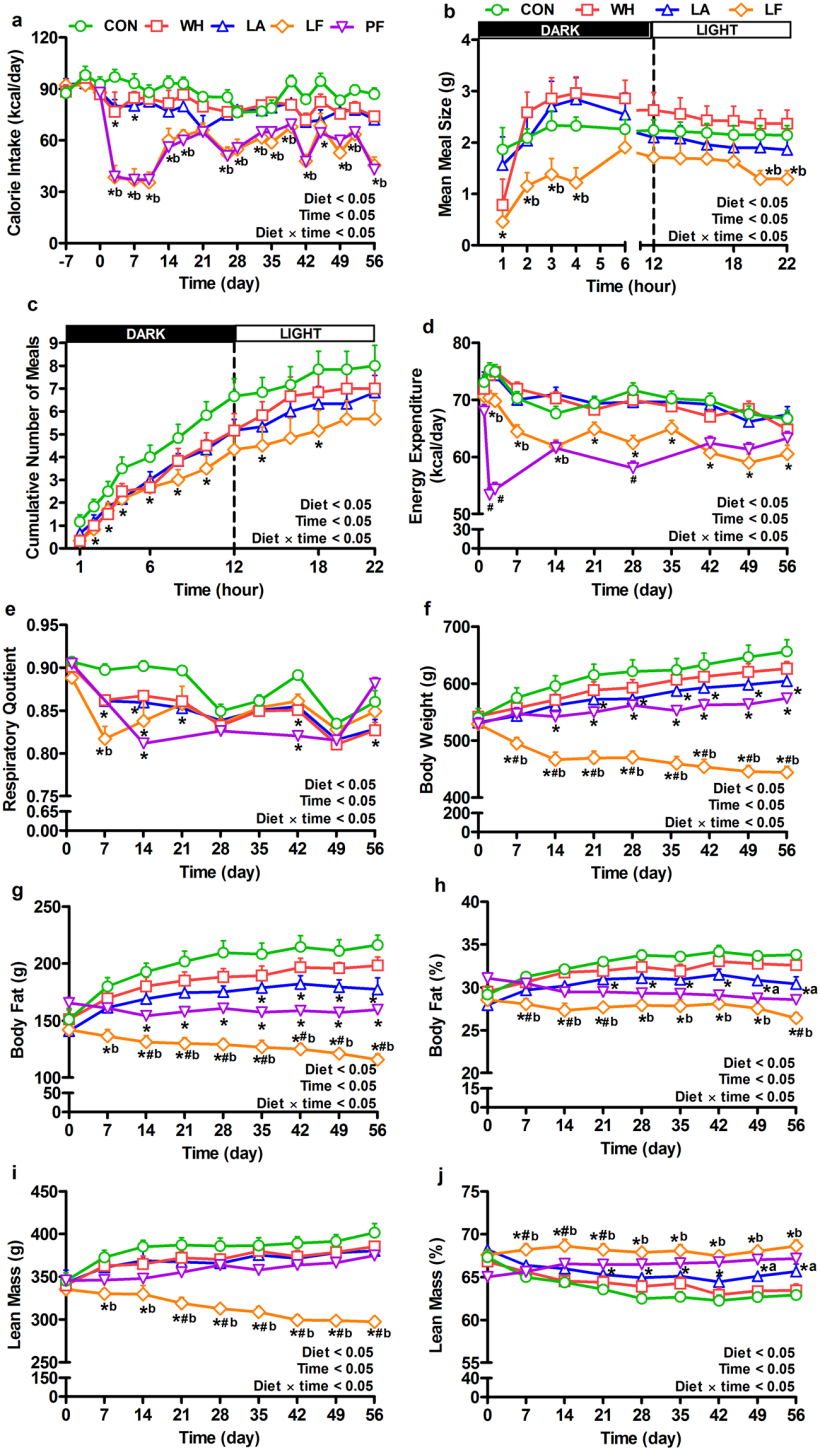
Effects of dietary whey, lactalbumin and lactoferrin on energy balance and body composition. (**a**) Calorie intake, (**b**) Mean meal size on day 2, (**c**) Cumulative number of meals on day 2, (**d**) Energy expenditure, (**e**) Respiratory quotient, (**f**) Body weight, (**g**) Body fat, (**h**) Percent body fat, (**i**) Lean mass and (**j**) Percent lean mass of diet-induced obese rats. Rats were fed either normal protein (control) diet (CON; green circles), or high protein diets enriched with whey protein isolate (WH; red square), α-lactalbumin (LA; blue triangle), lactoferrin (LF; orange diamond), or pair-fed (PF; inverted purple triangle) to LF, for 56 days. Energy expenditure was expressed as estimated mean of total heat production per day following ANCOVA with lean and fat mass as covariates. Values are expressed as mean ± SEM, n = 8/group. *P ≤ 0.05 vs CON, ^#^ *P* ≤ 0.05 LF vs PF, ^a^ *P* ≤ 0.05 LA vs WH, ^b^ *P* ≤ 0.05 LF vs WH.

We first compared the effects of diets enriched in whey protein, and its fractions - lactalbumin and lactoferrin, relative to a normal protein (control) diet, on food intake and meal patterns (Fig. 1a). Compared to control, whey decreased caloric intake by 10–21% for initial 4 days, lactalbumin by 14–23% for 7 days and lactoferrin by 20–68% for nearly the duration of the study (Fig. 2a). When compared to whey, lactalbumin did not decrease caloric intake, whereas lactoferrin reduced caloric intake by 13–62% for nearly the entire study (Fig. 2a) and decreased feed efficiency (Supplementary Fig. 1a). Meal pattern analyses on day 2 revealed that lactoferrin-induced hypophagia was partly due to decreased meal size during the initial dark period and decreased meal numbers throughout dark and light periods, whereas the hypophagic effects of whey and lactalbumin were only due to reduced meal number during the initial dark period (Fig. 2b,c).

### Lactoferrin attenuated the decrease in energy expenditure due to calorie restriction

Given the profound hypophagic effects of lactoferrin, and because caloric restriction often decreases energy expenditure, we determined the effect of whey fractions on energy expenditure and whether the reduced energy expenditure with lactoferrin was associated with hypophagia (Fig. 1a). Lactoferrin decreased energy expenditure by 6–13% compared to control for nearly throughout the experiment (Fig. 2d, Supplementary Fig. 1b). Lactalbumin induced marginally greater weight-normalized energy expenditure than control (Supplementary Fig. 1b). However, when compared to whey, lactalbumin did not alter energy expenditure whereas lactoferrin decreased expenditure by 5–17% during the first 3 weeks of the study (Fig. 2d, Supplementary Fig. 1b). Importantly, the energy expenditure of pair-fed animals was 7–24% lower than lactoferrin indicating that lactoferrin attenuated the calorie-dependent reduction in daily energy expenditure (Fig. 2d, Supplementary Fig. 1b). Whey, lactalbumin, lactoferrin and pair-fed decreased mean 24-h respiratory quotient (RQ) - suggestive of a shift towards lipid utilization with these diets (Fig. 2e).

### Lactoferrin decreased body weight and adiposity partly independent of calorie intake

We assessed whether the whey fraction-induced changes in energy intake and energy expenditure result in weight and fat loss, and whether such effects were independent of caloric intake (Fig. 1a). Compared to control, lactalbumin decreased body weight by 6–8% from day 21, and lactoferrin by 14–34% from day 7 onwards, whereas whey did not differ (Fig. 2f). Relative to control, lactalbumin decreased body fat by 14–19% from day 35 and lactoferrin by 24–48% from day 7 onwards (Fig. 2g). Although lactoferrin decreased lean mass by 11–26% from day 7 compared to control (Fig. 2i), the magnitude of this reduction was less than fat loss as percent lean mass was greater (Fig. 2j) and percent fat mass lower with lactoferrin (Fig. 2h). Despite similar body weights between lactalbumin and whey, lactalbumin decreased percent body fat (Fig. 2h) and increased percent lean mass (Fig. 2j) compared to whey. In addition, when compared to whey, lactoferrin decreased body weight by 21%, lean mass by 15% and body fat by 30%. Notably, the effects of lactoferrin on body composition were independent of food intake since lactoferrin decreased body weight, body fat and lean mass by 10–23%, 15–27% and 10–21%, respectively, compared to pair-fed from about day 7 onwards.

### Lactalbumin and lactoferrin-induced hypophagia were due to reduced preference

**Figure 3:**
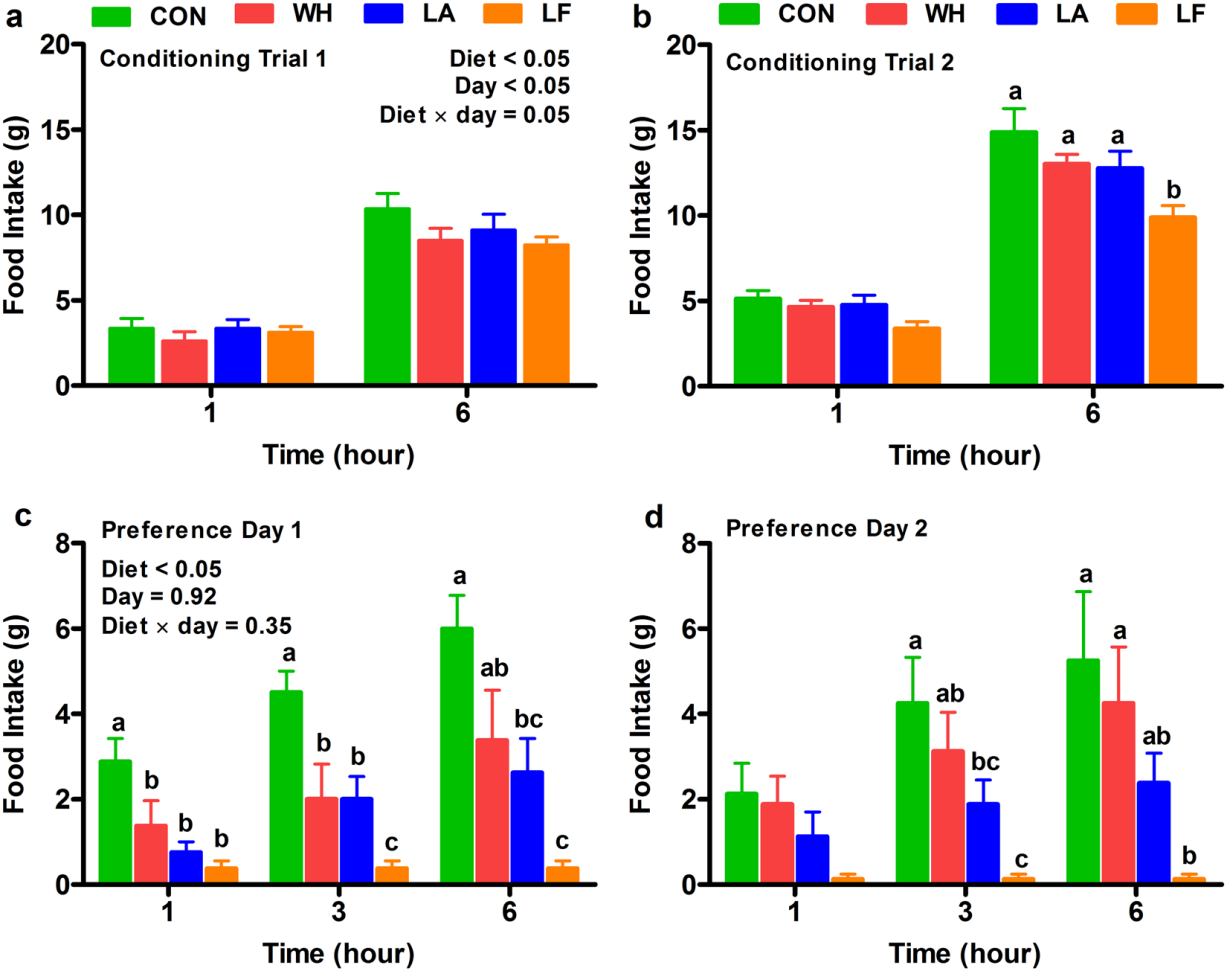
Effects of dietary whey, lactalbumin and lactoferrin on taste preference. Food intake during (**a**) Conditioning trial 1, (**b**) Conditioning trial 2, and (**c**) Day 1 and (**d**) Day 2 of preference tests. Diet-induced obese rats were fed normal protein (control) diet (CON; green bars), or high protein diets enriched with whey protein isolate (WH; red bars), α-lactalbumin (LA; blue bars) or lactoferrin (LF; orange bars). Values are expressed as mean ± SEM, n = 8/group. *P ≤ 0.05 vs CON; bars without a common letter differ.

Because the whey fractions decreased food intake, we determined in a separate cohort of rats whether the hypophagia was due to altered diet preference (Fig. 1b). Intakes of control, whey, lactalbumin and lactoferrin diets did not differ during the first conditioning trial (Fig. 3a). However, intake of lactoferrin was reduced during the second conditioning trial when compared to control (Fig. 3b). When given the choice of all four diets, preferences for lactalbumin and lactoferrin diets were decreased relative to control. The preferences for whey, lactalbumin and lactoferrin were decreased compared to control for the first three hours on the first day of preference test (Fig. 3c), and the decreased preference for lactoferrin persisted on the second day (Fig. 3d). Further, the preferences for whey and lactalbumin were decreased similarly, whereas lactoferrin was less preferred than whey on both days of preference test.

### Lactalbumin and lactoferrin improved glucose tolerance

**Figure 4:**
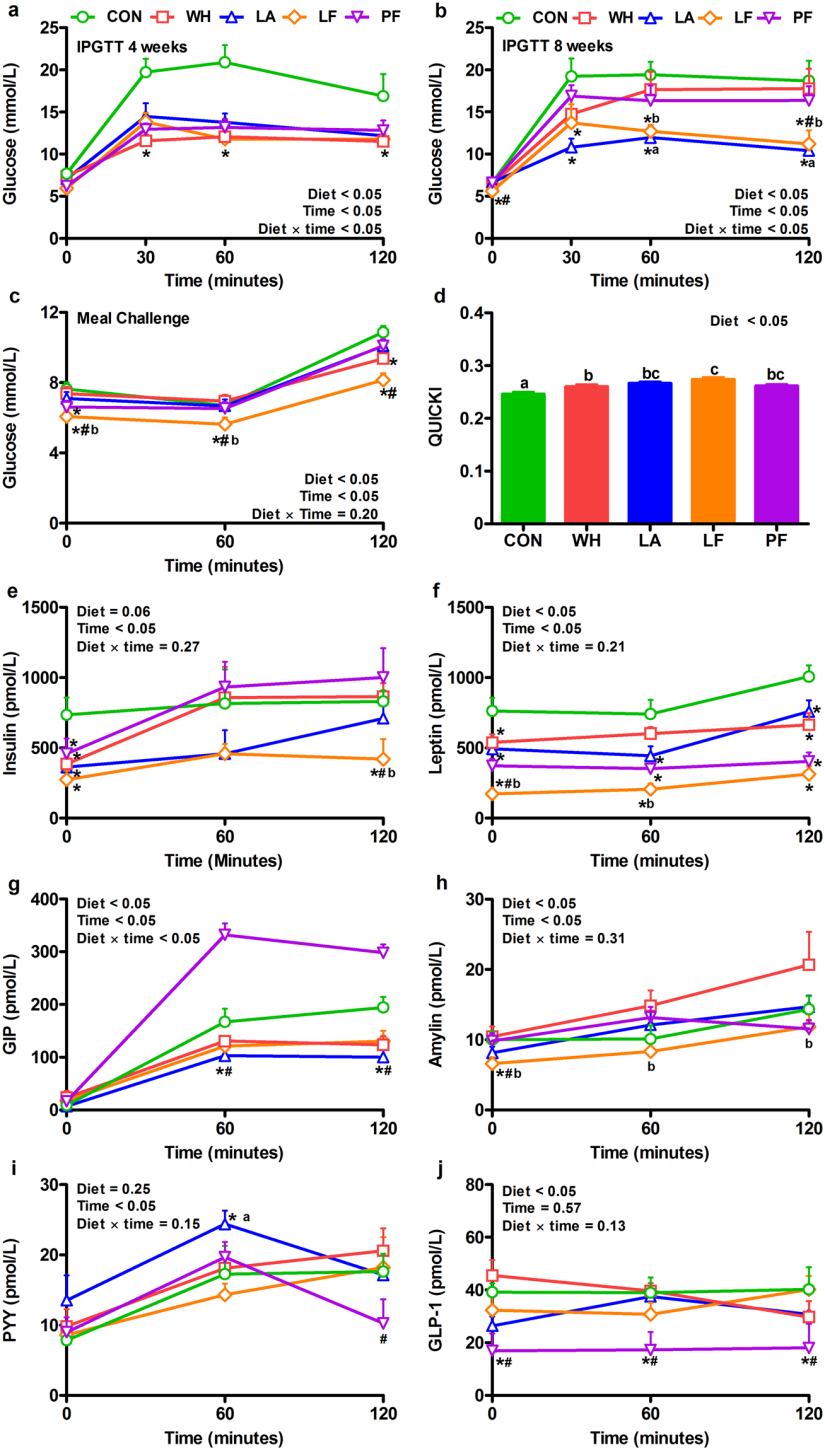
Effects of whey, lactalbumin and lactoferrin on circulating concentrations of glucose and hormones. Blood glucose concentrations after intraperitoneal glucose tolerance test (IPGTT) at (**a**) 4 weeks and (**b**) 8 weeks, (**c**) following a meal challenge, and (**d**) QUICKI. Plasma concentrations of (**e**) insulin, (**f**) leptin (**g**) glucose-dependent insulinotropic polypeptide (GIP), (**h**) amylin, (**i**) peptide YY (PYY) and (**j**) glucagon-like peptide-1 (GLP-1) after a meal challenge in diet-induced obese rats. Rats were fed either normal protein (control) diet (CON; green circles or bars), or high protein diets enriched with whey protein isolate (WH; red squares or bars), α-lactalbumin (LA; blue triangles or bars), lactoferrin (LF; orange diamond or bars) or pair-fed (PF; purple inverted triangles or bars) to LF, for 56 days. Values are expressed as mean ± SEM, n = 6–8/group. *P ≤ 0.05 vs CON, ^#^ *P* ≤ 0.05 LF vs PF, ^a^ *P* ≤ 0.05 LA vs WH, ^b^ *P* ≤ 0.05 LF vs WH, bars without a common letter differ.

We compared the effects of high protein diets containing whey fractions on glucose tolerance and assessed whether this was independent of caloric intake (Fig. 1a). The intraperitoneal glucose tolerance test (IPGTT) at 4 weeks revealed that whey, lactalbumin, lactoferrin and pair-fed significantly reduced blood glucose at 30, 60 and 120 min with a 35% reduction in area under the curve (AUC) compared to control (Fig. 4a, Supplementary Fig. 2a). However, IPGTT at 8 weeks showed that only lactalbumin and lactoferrin decreased blood glucose at 30, 60 and 120 min (Fig. 4b). Lactalbumin decreased glucose AUC by 32% compared to whey, and lactoferrin decreased glucose AUC by 27% compared to pair-fed, whereas whey and pair-fed did not differ from control (Supplementary Fig. 2b).

### Whey, lactalbumin and lactoferrin modulated meal-induced circulating glucose and anorexigenic hormone concentrations

Given the improvements in IPGTT, we determined whether high protein diets enriched with whey fractions altered meal-induced blood glucose and plasma concentrations of gut hormones and leptin when compared to a normal protein (control) diet (Fig. 1a). Lactoferrin decreased fasting and postprandial blood glucose concentrations compared to control or pair-fed, and lactalbumin decreased 120-min blood glucose compared to control (Fig. 4c). AUC analysis indicated that whey, lactalbumin, lactoferrin and pair-fed decreased postprandial blood glucose by 5%, 7%, 22% and 9%, respectively, compared to control, with lactoferrin decreasing glucose AUC by 17% than whey (Supplementary Fig. 2c). Compared to control, whey, lactalbumin, lactoferrin and pair-fed decreased fasting plasma insulin concentrations (Fig. 4e) and improved quantitative insulin sensitivity check index (QUICKI) (Fig. 4d). Further, lactoferrin decreased plasma insulin at 120 min compared to control, whey and pair-fed, and resulted in a greater improvement in QUICKI than whey and control (Fig. 4d,e). Whey, lactalbumin, lactoferrin and pair-fed decreased fasting, 60 and 120 min plasma leptin concentrations compared to control, and lactoferrin decreased fasting leptin relative to whey, lactalbumin and pair-fed (Fig. 4f). Fasting glucose-dependent insulinotropic peptide (GIP) concentrations did not differ among groups, but whey, lactalbumin and lactoferrin decreased circulating GIP at 60 and 120 min compared to control and pair-fed (Fig. 4g). Fasting amylin was also decreased with lactoferrin compared to control, whey and pair-fed, and at 60 and 120 min compared to whey (Fig. 4h). Compared to control, whey trended to increase amylin concentrations at 60 (P = 0.08) and 120 min (P = 0.09). Lactalbumin increased PYY concentrations at 60 min compared to control, whey and lactoferrin, while lactoferrin increased PYY at 120 min compared to pair-fed (Fig. 4i). In general, pair-feeding decreased plasma GLP-1 concentrations with no difference observed amongst other diets (Fig. 4j).

### Whey, lactalbumin and lactoferrin differentially modulated glucose and lipid metabolism in liver and skeletal muscle

**Figure 5:**
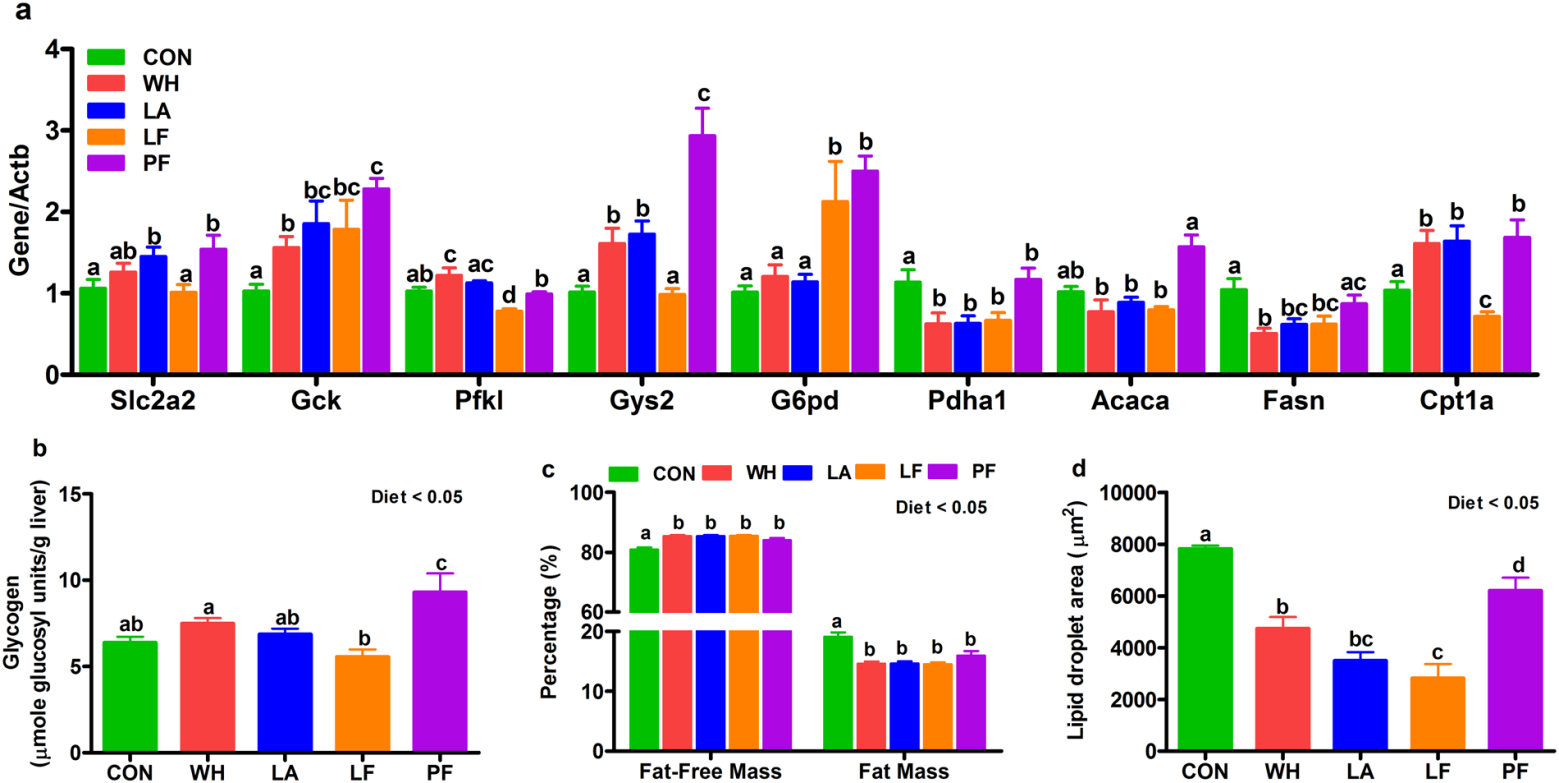
Effects of whey, lactalbumin and lactoferrin on liver glucose and lipid metabolism. The mRNA abundance of (**a**) glucose transporter-2 (Slc2a2), glucokinase (Gck), phosphofructokinase (Pfkl), glycogen synthase 2 (Gys2), glucose-6-phosphate dehydrogenase (G6pd), pyruvate dehydrogenase (Pdha1), acetyl-CoA carboxylase-1 (Acaca), fatty acid synthase (Fasn), and carnitine palmitoyltransferase-1 (Cpt1a) in the liver. (**b**) Total glycogen content, (**c**) liver composition and (**d**) area of lipid-laden zones of liver sections stained with hematoxylin-eosin are also shown. Rats were fed either normal protein (control) diet (CON; green bars), or high protein diets enriched with whey protein isolate (WH; red bars), α-lactalbumin (LA; blue bars), lactoferrin (LF; orange bars) or pair-fed (PF; purple bars) to LF, for 56 days. The relative mRNA abundance of each target was determined by qPCR with Actb as the house-keeping gene. Values are expressed as mean ± SEM, n = 5–8/group. Bars without a common letter differ.

**Figure 6:**
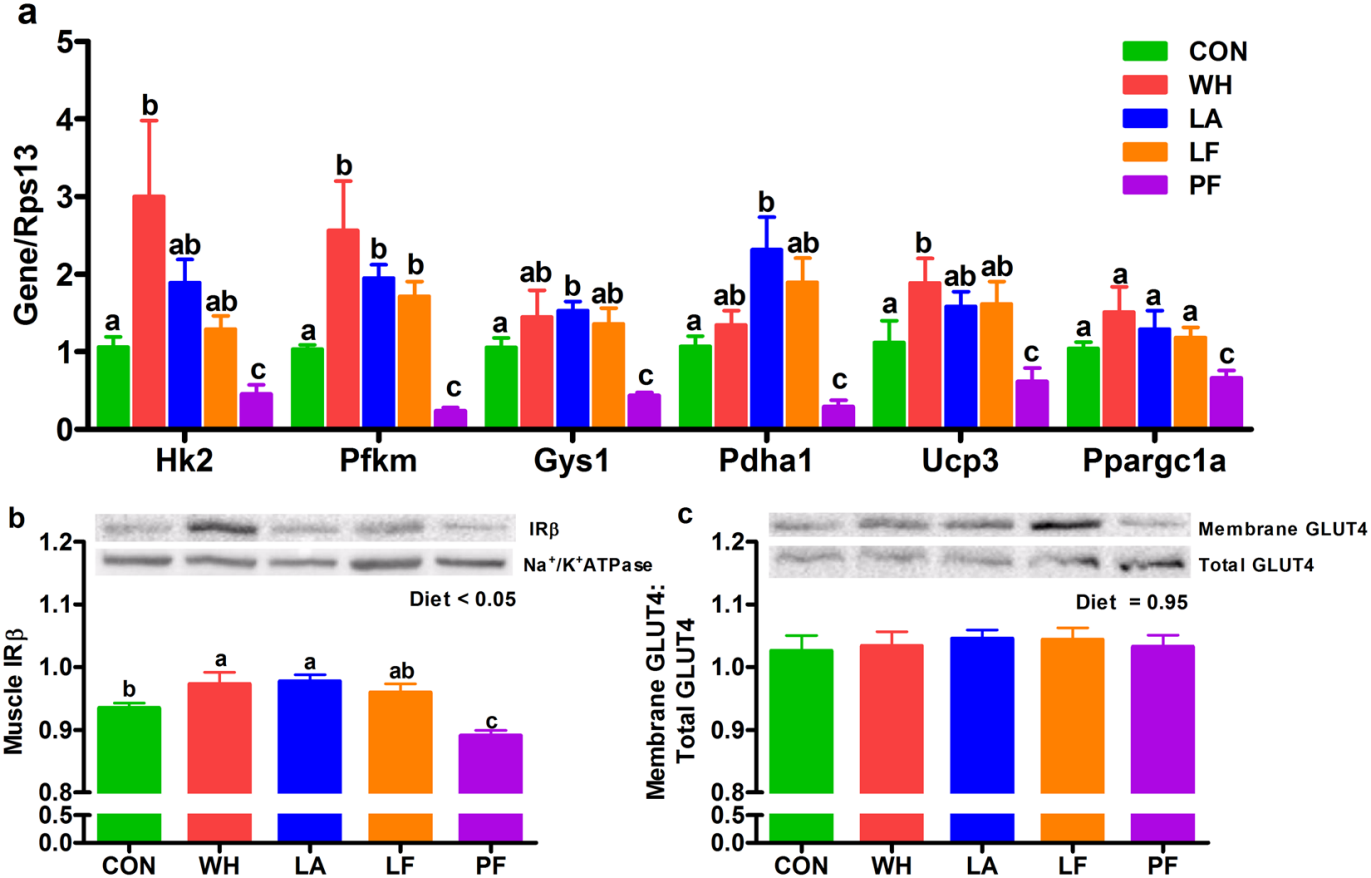
Effects of whey, lactalbumin and lactoferrin on glucose metabolism-related proteins and genes in skeletal muscle. The mRNA abundance of (**a**) hexokinase (Hk2), phosphofructokinase (Pfkm), glycogen synthase-1 (Gys1), pyruvate dehydrogenase (Pdha1), uncoupling protein-3 (Ucp3) and peroxisome proliferator-activated receptor gamma coactivator 1-alpha (Ppargc1a) in skeletal muscle. The relative protein abundance of (**b**) insulin receptor β-subunit (IRβ) and (**c**) GLUT4 in skeletal muscle is also shown. Rats were fed either normal protein (control) diet (CON; green bars), or high protein diets enriched with whey protein isolate (WH; red bars), α-lactalbumin (LA; blue bars), lactoferrin (LF; orange bars) or pair-fed (PF; purple bars) to LF, for 56 days. The relative mRNA abundance was determined by qPCR with 40 S ribosomal protein S13 (Rps13) as the reference gene, and the relative protein abundance was determined by immunoblotting with GAPDH or Na^+^/K^+^ATPase as loading controls. Values are expressed as mean ± SEM, n = 5–8/group. Bars without a common letter differ. Cropped blots are shown here and full-length blots are presented in Supplementary Fig. 4.

To gain insights into the mechanisms of glycemic improvements with whey fractions, we determined their effects on the mRNA and protein abundance of key regulatory molecules of glucose and lipid metabolism in the liver and muscle. In the liver, relative to control, lactalbumin increased the mRNA abundance of glucose transporter-2 (Slc2a2, Fig. 5a), whey and lactalbumin increased glucokinase (Gck, Fig. 5a), phosphofructokinase (Pfkl, Fig. 5a) and glycogen synthase-2 (Gys2, Fig. 5a). Interestingly, lactoferrin increased mRNA abundance of glucokinase (Gck, Fig. 5a) and glucose-6-phosphate dehydrogenase (G6pd, Fig. 5a), the rate-limiting enzyme of pentose phosphate pathway, compared to control, but decreased Slc2a2, Gck (P = 0.07), Pfkl, Gys2, Pdha1 and glycogen compared to pair-fed (Fig. 5a,b, Supplementary Fig. 3b). Whey and lactalbumin decreased hepatic lipid content (Fig. 5c,d, Supplementary Fig. 3a) partly by decreasing mRNA abundance of the lipogenic enzyme fatty acid synthase (Fasn, Fig. 5a) and upregulating the mRNA of carnitine palmitoyltransferase-1a (Cpt1a, Fig. 5a), the rate-limiting enzyme for mitochondrial fatty acid uptake, compared to control. Compared to pair-fed, lactoferrin decreased the hepatic lipid content (Fig. 5c,d) and mRNA abundance of acetyl-CoA carboxylase-1 (Acaca, Fig. 5a), Fasn (P = 0.07, Fig. 5a) and Cpt1a (Fig. 5a). In the muscle, compared to control, whey and lactalbumin increased IR-β protein (Fig. 6b) but the ratio of membrane-bound to total GLUT4 remained unchanged (Fig. 6c). Lactalbumin tended to increase mRNA abundance of hexokinase (Hk2, Fig. 6a) and significantly increased phosphofructokinase (Pfkm, Fig. 6a) and glycogen synthase 1 (Gys1, Fig. 6a), while whey increased Hk2 and Pfkm (Fig. 6a). Relative to pair-fed, lactoferrin increased the protein abundance of muscle IR-β and transcripts for Hk2, Gys1 and Pdha1 (Fig. 6a). In addition, compared to pair-fed, lactoferrin increased the transcripts for uncoupling protein-3 (Ucp3, Fig. 6a) and peroxisome proliferator-activated receptor gamma coactivator 1-alpha (Ppargc1a, Fig. 6a), important regulators of lipid oxidation and thermogenesis.

### Lactalbumin and lactoferrin differentially modulated cecal bacterial populations

**Figure 7:**
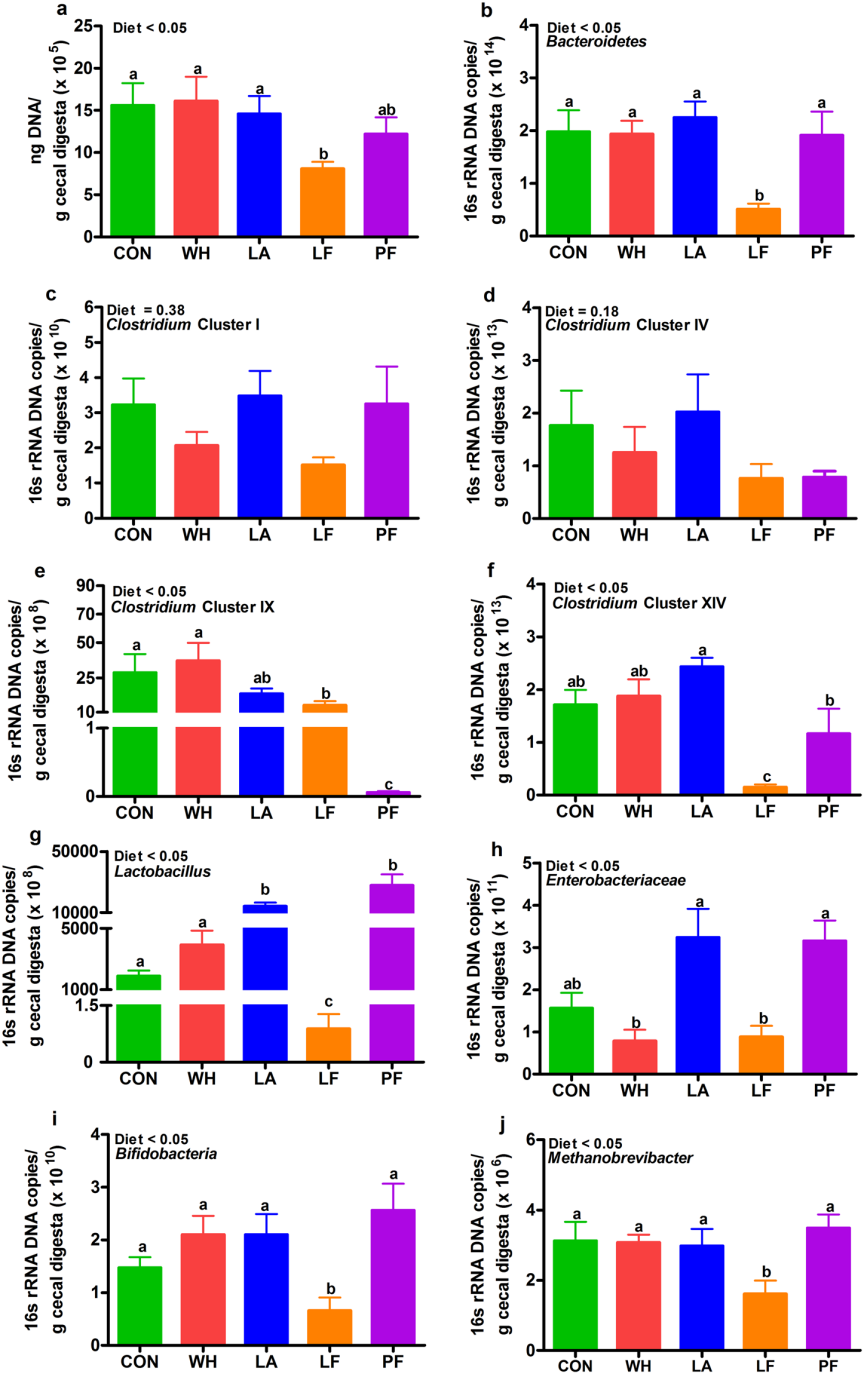
Effects of whey, lactalbumin and lactoferrin on numbers of select cecal bacterial populations. (**a**) DNA yield/g cecal digesta, and 16 S rRNA gene copies/g cecal digesta of (**b**) *Bacteroidetes*, (**c**) *Clostridium* Cluster I, (**d**) *Clostridium* Cluster IV, (**e**) *Clostridium* Cluster IX, (**f**) *Clostridium* Cluster XIV, (**g**) *Lactobacillus*, (**h**) *Enterobacteriaceae*, (**i**) *Bifidobacterium*, (**j**) *Methanobrevibacter*. Rats were fed either normal protein (control) diet (CON; green bars), or high protein diets enriched with whey protein isolate (WH; red bars), α-lactalbumin (LA; blue bars), lactoferrin (LF; orange bars) or pair-fed (PF; purple bars) to LF, for 56 days. The 16 S rRNA gene copies were quantified by qPCR. Values are expressed as mean ± SEM, n = 6–8/group. Bars without a common letter differ.

As little is known of the effects of whey fractions on gut microbiota, we quantified the abundance of select bacteria in the cecum in response to dietary interventions. The cecal bacterial 16 S rRNA gene copies for most strains were comparable between whey and control. Lactalbumin increased the 16 S rRNA gene copies of *Lactobacillus* (Fig. 7g) and *Clostridium* cluster XIV (*P* = 0.09; Fig. 7f). Interestingly, lactoferrin decreased the total DNA yield/g of cecal digesta suggestive of inhibition of the growth of most bacterial populations (Fig. 7a,b,e–j) except *Clostridium* cluster I and *Clostridium* cluster IV (Fig. 7c,d). The effects of lactoferrin on cecal bacterial population were independent of calorie intake since pair-fed increased bacterial 16 S rRNA gene copies of most bacterial populations studied (Fig. 7b,f–j) except *Clostridium* clusters I, IV and IX (Fig. 7c–e).

## Discussion

The health benefits of dietary whey protein on obesity and diabetes has been well documented in animal models and humans^9, 10, 35, 36^, yet, less is known of the relative efficacies of whey protein fractions lactalbumin and lactoferrin, and their underlying mechanisms of action. Here, we compared the effects of normal protein (control) diet with high protein diets containing whey, or its fractions lactalbumin and lactoferrin, on energy balance and metabolism. First, relative to control, whey and lactalbumin transiently decreased food intake, whereas, lactoferrin induced prolonged hypophagia compared to control and whey. The hypophagic effects of the whey components may in part be due to decreased dietary preference. Second, unlike whey, lactalbumin and lactoferrin decreased weight gain and fat mass compared to control. Importantly, lactoferrin produced sustained weight and fat loss, which is in part due to an attenuation of the reduction in energy expenditure that accompanied caloric restriction. Third, lactalbumin and lactoferrin produced sustained reductions in glucose excursions than control, with the glycemic improvements of lactoferrin being partly independent of caloric intake. Interestingly, whey and lactalbumin improved glucose clearance after a meal partly through upregulation of glycogenic genes in the liver and muscle, whereas, lactoferrin upregulated pentose-phosphate pathway genes. Fourth, lactalbumin and lactoferrin decreased hepatic lipidosis, relative to control, partly through downregulation of lipogenic and/or upregulation of β-oxidation transcripts in the liver. And fifth, whey, lactalbumin and lactoferrin differentially modulated the plasma concentrations of anorexigenic hormones, as well as the abundance of specific cecal bacterial populations that were previously shown to be associated with improved energy balance and glycemia. Taken together, these data provide evidence that lactalbumin and lactoferrin are more effective than whey in decreasing weight and adiposity, as well as in improving glycemic control in diet-induced obese rats, with the effects of lactoferrin extending beyond caloric restriction.

There is substantial evidence that diets high in protein acutely decrease food intake^1^, however, less is known of their chronic effects on food intake and the underlying mechanism of action. We demonstrate that dietary whey, lactalbumin and lactoferrin produce hypophagia of different magnitude and duration with the effects of whey and lactalbumin being transient for a few days, whereas lactoferrin induced robust and prolonged hypophagia. Consistent with our findings, dietary lactalbumin was reported to decrease intake in rats^37^ and, whey and lactalbumin-supplemented breakfasts were reported to reduce hunger, desire to eat^15^ and decrease caloric intake^33^ in humans. Daily supplementation of lactoferrin at 100 mg did not affect energy intake in humans^27^, and lactoferrin at 3%^24^ or 20%^25^ of total calories also did not decrease food intake in mice. In contrast, we report that lactoferrin at 15% of total calories led to a greater and prolonged hypophagia compared to control, whey and lactalbumin. The hypophagic effects of lactoferrin were also reproducible during acute conditioning trials. Given the decreased preference for diets enriched with whey, lactalbumin and lactoferrin, it is likely that reduced palatability may play a role in the initial hypophagic effects of whey and lactalbumin as we^9, 10^ and others^37^ have previously reported; however, whether altered preference persists chronically, especially with lactoferrin, remains to be determined. The hypophagic effects of whey-based diets are often associated with enhanced secretion of lower gut satiety hormones. For example, dietary whey^29–31^, but not lactalbumin^16, 21, 33^, has been shown to acutely increase plasma concentrations of GLP-1, GIP and/or PYY in humans^29–31^. We and others previously reported that whey chronically increased plasma GLP-1 in rats^9, 32^, that lactalbumin increased GLP-1 secretion from an enteroendocrine cell line^38^, and that systemic lactoferrin acutely increased GLP-1 in rats^39^; however, less is known of the relative effects of whey fractions on gut hormone secretion in the long-term. Importantly, for the first time, we report that dietary whey components differentially influence the secretion of gut satiety hormones: whey and lactalbumin increased plasma amylin and PYY concentrations, respectively, without alterations in GLP-1. It remains to be determined whether altered secretions of these satiety hormones are causative to the hypophagic effects of the diets.

The effects of dietary whey components in modulating energy expenditure are poorly understood. Consumption of foods enriched with whey or lactalbumin increased energy expenditure compared to casein or soy in some human studies^15, 40^ but not others^21, 41^. Further, dietary lactalbumin was found to acutely increase energy expenditure in exercising rats^34^; however, the long-term adaptations in energy expenditure following consumption of whey components are unknown. Here, whey did not alter energy expenditure despite a transient hypophagia, and lactalbumin increased weight-normalized energy expenditure; however, whether these diet interventions acutely alter energy expenditure remains to be determined. In contrast, lactoferrin decreased intake, attenuated the decrease in expenditure accompanying calorie restriction, induced thermogenic genes in the muscle and produced greater weight and fat loss than pair-fed, despite both lactoferrin and pair-fed groups consuming identical amount of calories. Taken together, these findings indicate that the effects of lactoferrin on energy expenditure are in part due to caloric restriction, but more importantly, also beyond simply the amount of calories consumed. Thus, the greater loss of weight and lean mass with lactoferrin could likely be due to thermogenesis, decreased feed efficiency and/or potential fecal energy losses. Of note, the magnitude and duration of weight loss with lactoferrin-enriched diets shown here were comparable to our previous findings on the weight loss effects of Roux-en-Y gastric bypass surgery in rats^42^. Though whey (15% calories) was ineffective in sustaining weight loss here than in our previous study (25% calories)^9^, it is noteworthy that lactalbumin, which produced similar degree of hypophagia as whey, was more effective than whey in decreasing adiposity. These findings highlight a role for whey protein components, lactoferrin in particular, in reducing weight and adiposity and indicate that the effects of lactoferrin were in part independent of calorie intake.

The initial improvements in glucose tolerance, and meal-induced glucose clearance, with whey were consistent with our previous study^9^. We now show that such early improvements with whey were independent of weight and fat loss since body weight and composition were comparable between control and whey. However, similar to a previous study^17^, long-term improvements in glucose tolerance were only sustained with lactalbumin and lactoferrin and were associated with decreased adiposity, plasma insulin and leptin concentrations. The reduction in GIP with lactalbumin, and lack of changes in GLP-1, suggest that these incretins were unlikely to play a role in the glycemic improvements. Others reported that whey increases muscle glycogenesis by upregulating glycogen synthase in mice^11^. We demonstrate here that whey and lactalbumin promoted hepatic glycogenesis by upregulating transcripts for glucose metabolism (Gck) and glycogen synthesis (Gys2). In contrast to pair-fed, lactoferrin downregulated majority of these transcripts (Slc2a2, Pfkl, Gys2) suggestive of calorie-independent effects in improving glucose metabolism. Similar to the liver, in the muscle, whey and lactalbumin upregulated key regulatory molecules of glucose metabolism (IR-β, Pfkm) while lactoferrin-induced changes in glucose metabolism related genes (Hk2, Pfkm, Gys1, Pdha1) were independent of caloric intake. Previous reports showed that whey^12, 13^, lactalbumin^12^ and lactoferrin^23^ decrease hepatic lipidosis partly by downregulating lipogenic and/or upregulating lipolytic genes in rodents^13^. We expand on these findings by demonstrating that whey, lactalbumin and lactoferrin decreased hepatic lipidosis partly by decreasing lipogenic transcripts (Fasn), with whey and lactalbumin also upregulating the transcripts of fatty acid oxidation (Cpt1a). Unlike pair-fed, lactoferrin decreased hepatic lipidosis by downregulating lipogenic transcripts (Fasn, Acaca) indicating that the effects of lactoferrin are independent of caloric intake. Thus, our findings suggest that whey and lactalbumin enhanced glucose clearance and metabolism partly through upregulation of glycogenic genes in the liver and muscle, lactoferrin through increased pentose-phosphate pathway transcripts, while both lactalbumin and lactoferrin decreased hepatic lipidosis partly through decreased lipogenic and/or enhanced β-oxidation transcripts.

Lactoferrin has been reported to have antimicrobial properties through its ability to chelate iron, disrupt microbial membranes and prevent bacterial biofilm formation^43, 44^. We demonstrate that lactoferrin decreased most cecal bacteria independent of calorie restriction. In contrast, others reported that lactoferrin supplementation promotes the growth of *Bifidobacteria* ^22, 45^, *Lactobacillus* ^45^ and *Bacteroidetes* ^46^ but inhibits *Enterobacteriales* ^45, 46^. The disparities could likely be due to differences in animal models and dosage. Our diets were designed to be isonitrogenous with lactoferrin intakes at ~4 g/kg BW/day (15% calories) which was lower than a previous report on feeding 20% lactoferrin to mice^25^. In contrast, others supplemented lactoferrin at lower doses of 100 mg/kg BW/day in mice^22^ or 1.5 g/kg BW/day in piglets^45^. At the relatively high doses in our study, lactoferrin may have antimicrobial properties to produce a lean and glucose tolerant phenotype^47^. We also show that lactalbumin increased *Lactobacillus* and butyrate-producing bacteria (*Clostridium* cluster XIV), which are both associated with weight loss and improved insulin sensitivity^48, 49^.

In conclusion, our findings highlight a previously unappreciated role for high protein diets enriched with whey protein components - lactalbumin and lactoferrin - in improving energy balance, glucose and lipid metabolism, and in modulating gut microbiota and circulating gut satiety hormone concentrations. Importantly, we demonstrate that the reduction of adiposity and improvements in glucose metabolism by lactoferrin are beyond calorie intake.

## Methods

### Experimental animals

Experimental protocol (AC12-0033) was approved by the University of Calgary Animal Care and Use Committee, and all experiments were performed with the relevant guidelines and regulations. Male obese prone (OP-CD) rats (6-week old, 168 ± 3 g; Charles River, Montreal, QC, Canada) were individually housed and acclimatized to the environmental conditions for a week (12:12-hour light-dark cycle, dark-onset 1030 h, temperature 23–25 °C, humidity 21–22%). Obesity was induced by a high fat diet (40% fat, 15% protein, 45% carbohydrate calories) for ~8 weeks. Two experiments were conducted on separate cohorts of obese rats with *experiment-1* focusing on the effects of normal and high protein diets enriched with whey fractions on long-term changes in energy balance, and *experiment-2* on diet preferences of the obese rats in the short-term. To determine the effects of both protein quantity and quality on energy balance and metabolism, in *experiment-1* (Fig. 1a), obese rats (536 ± 7 g, 14 weeks of age, n = 8/group) were randomized to receive powdered isocaloric high fat diets (4.63 kcal/g; Table 1): (1) Control (15% egg albumen), (2) Whey (15% whey protein isolate + 15% egg albumen), (3) Lactalbumin (15% α-lactalbumin + 15% egg albumen), (4) Lactoferrin (15% lactoferrin + 15% egg albumen) or (5) Pair-fed to lactoferrin (15% whey protein isolate + 15% egg albumen) for 56 days. Water was freely available and, except pair-fed, all rats were fed *ad libitum*. In a pilot study, we found that rats consumed negligible amount of food when lactoferrin was included at 30% of total calories. To avoid confounds of profound lactoferrin-induced anorexia, while maintaining total dietary protein calories at 30%, egg albumen was included at a basal 15% of total calories in all diets. To determine whether the effects of lactoferrin on energy balance and glucose metabolism were independent of caloric intake, and to account for the protein content of lactoferrin, the average daily food intake of lactoferrin fed rats was calculated and this amount was fed to the pair-fed rats with the isonitrogenous whey diet, to ensure that both lactoferrin and pair-fed rats received similar total calories and protein.

**Table 1:** Diet Composition.

Ingredients	Control	Whey	Lactalbumin	Lactoferrin
Corn Starch^1^	374	200	200	200
Sucrose^1^	147.8	147.8	147.8	147.8
Egg Albumen^1^	174	174	174	174
Whey Protein Isolate^2^	0	174	0	0
Lactalbumin^3^	0	0	174	0
Lactoferrin^2^	0	0	0	174
Corn Oil^4^	60	60	60	60
Lard^5^	145	145	145	145
α- cellulose^1^	50	50	50	50
AIN-93-MX^1^	35	35	35	35
AIN-93-VM^1^	10	10	10	10
L-cystine^1^	1.8	1.8	1.8	1.8
Choline bartartrate^1^	2.5	2.5	2.5	2.5
*Macronutrients* ^6^:				
Protein (% kcal)	15%	30%	30%	30%
Carbohydrate (% kcal)	45%	30%	30%	30%
Fat (% kcal)	40%	40%	40%	40%
Total kcal/g	4.63	4.63	4.63	4.63

^1^Dyets Inc. (Bethlehem, PA, USA), ^2^whey protein isolate (91.3% protein, 2.67% ash, 4.8% moisture), lactoferrin (92.1% protein, 1.05% ash, 3.26% moisture) Advanced Orthomolecular Research (Calgary, AB, Canada), ^3^α-lactalbumin (93.6% protein, 0.6% ash, 3.88% moisture) MP Biomedicals (Solon, OH, USA), ^4^La Perla (Montreal, QC, Canada), ^5^Sunspun® (Toronto, ON, Canada), ^6^Energy density was calculated from the calorific values of protein, fat and carbohydrate at 4, 9 and 4 kcal/g respectively.

### Food Intake, Energy Expenditure and Body Composition

Daily food intake, meal patterns and energy expenditure were measured throughout the study using a 32-unit Oxymax/CLAMS® (Columbus Instruments, Columbus, OH) as previously described^9^. All treatment group animals were acclimatized to the CLAMS system for about a week on control diet prior to measurements. However, due to space constraints, the measurements of PF group were restricted to days 0–3, 14–17, 30–31, 44–46, 49–51, 53–56 of the study. Meal patterns were defined using a minimum meal size of 50 mg and intermeal interval of 15 min^50^. Body weight was measured bi-weekly and body composition weekly using Minispec LF-110® (Bruker Optics, Billerica, MA).

### Glucose Tolerance, Meal Challenge and Hormone Analyses

An IPGTT was performed at 4 weeks (day 27–30) and 8 weeks (day 48–51) as previously described^51^. Briefly, on any given test day, two rats from each group were fasted overnight (~16 h) and then received intraperitoneal injections of 50% dextrose (2 g/kg BW) followed by blood glucose measurements at 0, 30, 60 and 120 min using a glucometer (Roche Diagnostics®, Laval, QC, Canada). For the meal challenge, at day 55–58, animals were fasted overnight and then provided access to their respective diets for 60 min. Blood was sampled at 0, 60 and 120 min after meal onset and glucose concentrations measured. Plasma samples were processed and skeletal muscle, liver and cecal digesta collected at 120 min after meal onset, snap-frozen in liquid nitrogen and stored at −80 °C as previously described^9^. Hormone assays for insulin, leptin, GIP, amylin and PYY were performed by Eve Technologies (Calgary, AB, Canada) using Luminex® Multiplex assay (EMD Millipore, Billerica, MA) and AUCs calculated. The intra- and inter-assay CV’s were 3.8% and 9.0% for insulin, 5.9% and 1.4% for leptin, 4.8% and 10.4% for GIP, 8.6% and 18.5% for amylin and 5.0% and 13.7% for PYY, respectively. Fasting glucose and insulin concentrations were used to calculate QUICKI^52^. The food intake and energy expenditure data from the test animals, from the day before to the day after the glucose tolerance tests, were excluded from the data analyses.

### Hepatic lipid and glycogen analyses

Liver biopsies were analyzed for fat content using a tissue probe in Minispec® LF-110, and glycogen measured as previously described^53^. Liver lipid content was estimated in 10% formalin-fixed hematoxylin-eosin stained liver sections using ImageJ® (NIH, Bethesda, MD). Liver glycogen was visualized using periodic acid-Schiff stain.

### Tissue qRT-PCR and Immunoblotting

The qRT-PCR and immunoblotting of glucose, lipid and thermogenesis-related markers were performed on muscle and liver following our published protocols^9, 54^ using target primers and antibodies (Supplementary Tables 1 and 2). Briefly, RNA was extracted from tissues with QIAzol and purified using RNeasy® Lipid Tissue Mini Kit (#74804, Qiagen®, Toronto, ON, Canada). Total RNA (31.25 ηg; 20 μl) was reverse-transcribed using Superscript II® (Life Technologies®, Carlsbad, CA) followed by PCR using Power SYBR Green PCR Master Mix and 100 μM primers, in duplicate, on a Mastercycler ep Realplex thermocycler (Eppendorf Canada, Mississauga, ON). Relative quantification of the target gene was calculated by the 2^−∆∆CT^ method, in reference to control diet group, with Actb (liver) and Rps13 (muscle) as housekeeping genes. Immunoblotting was performed on 25 μg of muscle protein extracts which were fractioned using a 10% SDS gel, transferred onto nitrocellulose membranes, blocked with skim milk, incubated with primary antibodies and subsequently detected with fluorescent secondary antibodies using a ChemiDoc MP® imaging system (Bio-Rad Laboratories Ltd., Mississauga, ON, Canada). The band intensity of each target protein was normalized to either GAPDH (total GLUT4) or Na^+^/K^+^-ATPase (membrane bound IR-β and GLUT4).

### Cecal bacterial DNA isolation and qPCR

Bacterial DNA was extracted from cecal digesta using QIAamp® DNA Stool Kit (Qiagen®, Toronto, Canada). Briefly, ~200 mg of pulverized cecal contents were suspended in 1.4 ml of ASL lysis buffer, incubated at 70 °C for 5 min, DNA purified according to the manufacturer’s prescribed protocols, and stored at −80 °C. DNA was quantified using Quant-iT® PicoGreen dsDNA assay kits (ThermoFisher Scientific®, Eugene, OR). Quantitative PCR was performed using Power SYBR Green PCR mastermix, 100 nM of each primer and 20 ηg of DNA in a 25 µl reaction. 16 S rRNA gene copies from different bacterial groups were amplified using specific primers^55^ (Supplementary Table 3). Standard curves were generated using serial ten-fold dilutions of bacterial DNA (DSMZ, Braunschweig, Germany). The 16 S rRNA gene copies/g of cecal digesta was calculated using the following formula: 16 S rRNA gene copies = [(amount of DNA template × 6.022 × 10^23^)/(base pairs × (1 × 10^9^) × 660)] × DNA yield.

### Diet Preference

To test whether the reductions in food intake with whey, lactalbumin and lactoferrin diets were due to altered diet preference, in *experiment-2* (Fig. 1b), preference tests were conducted on a separate cohort of naïve obese OP-CD rats (n = 8, ~12 weeks of age, 413 ± 6 g) following modifications of our previous protocols^9, 56^. Briefly, to minimize potential neophobia to a diet, two consecutive conditioning trials were conducted over 8 days followed by two days of preference testing. In each conditioning trial, on alternate days, rats had 6 h access to either powdered control, whey, lactalbumin and lactoferrin with 4% grape-flavored KoolAid® to mask any inherent flavors. Subsequently, the rats had *ad libitum* access to powdered chow (Picolab® 5053, Labdiet®, St. Louis, MO) to minimize any carry-over effects of test diets. After two periods of conditioning, two days of preference tests were conducted with rats having simultaneous access to all four diets. To minimize feeder bias, the positions of the feeding jars were changed after each food intake measurement.

### Statistics

Repeated measures on food intake, meal size and number, energy expenditure, respiratory quotient, body weight and composition, and blood glucose post-IPGTT were analyzed by linear mixed models (IBM SPSS® v20) using fixed effects of diet, time (hour, day), drug, and their interactions, as appropriate. Animal nested in the group was the random variable on which repeated measures were taken and appropriate covariance structure selected based on the smallest values of AIC and BIC criteria. Energy expenditure was analyzed by ANCOVA using weekly measures of lean and 20% of fat mass as covariates^57^. Blood glucose and hormone concentrations post-meal were analyzed by ANCOVA with food consumed as covariate. The mRNA and protein abundance, and cecal microbial 16 S rRNA gene copies were analyzed using one-way ANOVA. Fisher’s least significant difference posthoc test was used to determine group differences. Significance was set at *P* ≤ 0.05 and trends at *P* ≤ 0.10.

### Data availability

The datasets generated during and/or analysed during the current study are available from the corresponding author on reasonable request.

## Electronic supplementary material

Supplementary Information
