# Supplementary material for: Whey Protein Components - Lactalbumin and Lactoferrin - Improve Energy Balance and Metabolism
Source: Sci Rep. 2017 Aug 30;7:9917. doi: 10.1038/s41598-017-09781-2 (PMC5577213; doi:10.1038/s41598-017-09781-2)
Supplement: Supplementary file 1 — Supplementary Information [file 41598_2017_9781_MOESM1_ESM.pdf]

Supplementary Information

Whey Protein Components - Lactalbumin and Lactoferrin - Improve Energy Balance and Metabolism

Rizaldy C. Zapata[1], Arashdeep Singh[1], Adel Pezeshki[2], Traj Nibber[3], Prasanth K. Chelikani[1,4 *]

[1] Department of Production Animal Health, Faculty of Veterinary Medicine, University of Calgary, 3330 Hospital Drive NW, Calgary, Alberta, Canada, T2N 4N1

[2] Department of Animal Science, Oklahoma State University, Stillwater, OK, USA, 74078

[3] Advanced Orthomolecular Research, 3900 12 St NE, Calgary, Alberta, Canada, T2E 6X8

[4] Gastrointestinal Research Group, Snyder Institute of Chronic Diseases, University of Calgary, 3330 Hospital Drive NW, Calgary, Alberta, Canada, T2N 4N1

*Corresponding Author:

Prasanth K. Chelikani, BVSc, MVSc, PhD

HS 1871, 3330 Hospital Drive NW, Calgary, Alberta, Canada, T2N 4N1

Tel: +1 (403) 210-7652; Fax: +1 (403) 210-6693

Email: pchelika@ucalgary.ca

Supplementary Information

Supplementary Figure 1. Effects of whey, lactalbumin and lactoferrin on feed efficiency and energy expenditure. (**a**) Feed efficiency and (**b**) daily energy expenditure normalized to body weight. Feed efficiency was expressed as the ratio of weekly weight gain to the amount of food consumed in gm, and energy expenditure as the ratio of total heat produced (kcal) to body weight (kg). Rats were fed either normal protein (control) diet (CON; green circles), or high protein diets enriched with whey protein isolate (WH; red square), α-lactalbumin (LA; blue triangle), lactoferrin (LF; orange diamond), or pair-fed (PF; inverted purple triangle) to LF, for 56 days. Values are expressed as mean ± SEM, n = 8/group. *$P \leq 0.05$ vs CON, $^{\#}P \leq 0.05$ LF vs PF, $^{b}P \leq 0.05$ LF vs WH.

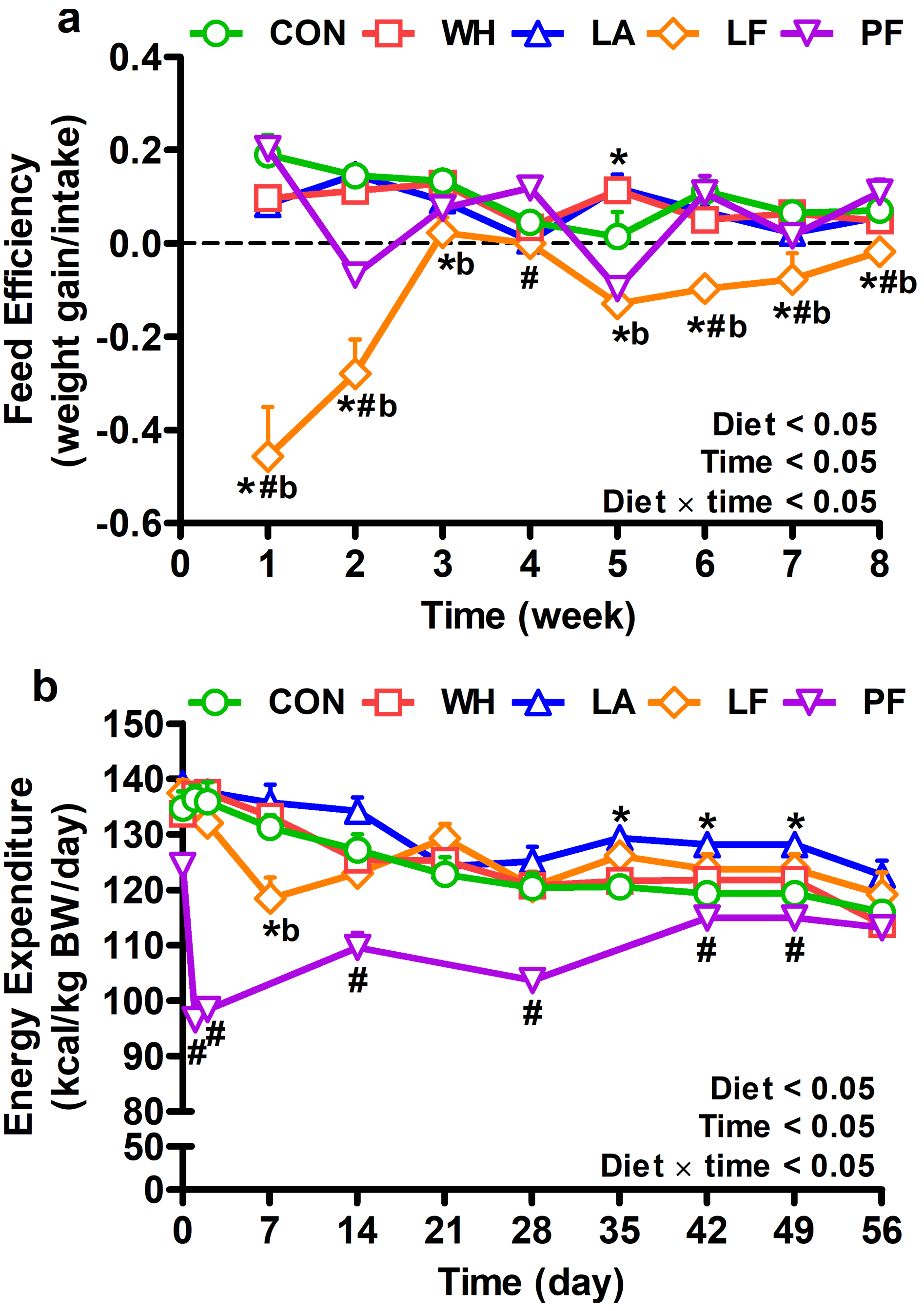

Supplementary Information

Supplementary Figure 2. Effects of whey, lactalbumin and lactoferrin on plasma hormones. Area-Under-the-Curve analyses for (**a**) IPGTT 4 weeks, (**b**) IPGTT at 8 weeks, (**c**) blood glucose following meal challenge, and plasma concentrations of (**d**) insulin, (**e**) leptin, (**f**) glucose-dependent insulinotropic polypeptide (GIP), (**g**) amylin, and (**h**) peptide YY (PYY), and (**i**) glucagon-like peptide-1 (GLP-1) following a meal challenge at 8 weeks. Rats were fed either normal protein (control) diet (CON; green bars), or high protein diets enriched with whey protein isolate (WH; red bars), α-lactalbumin (LA; blue bars), lactoferrin (LF; orange bars) or pair-fed (PF; purple bars) to LF, for 56 days. Values are expressed as mean ± SEM, n = 8/group. Bars without a common letter differ.

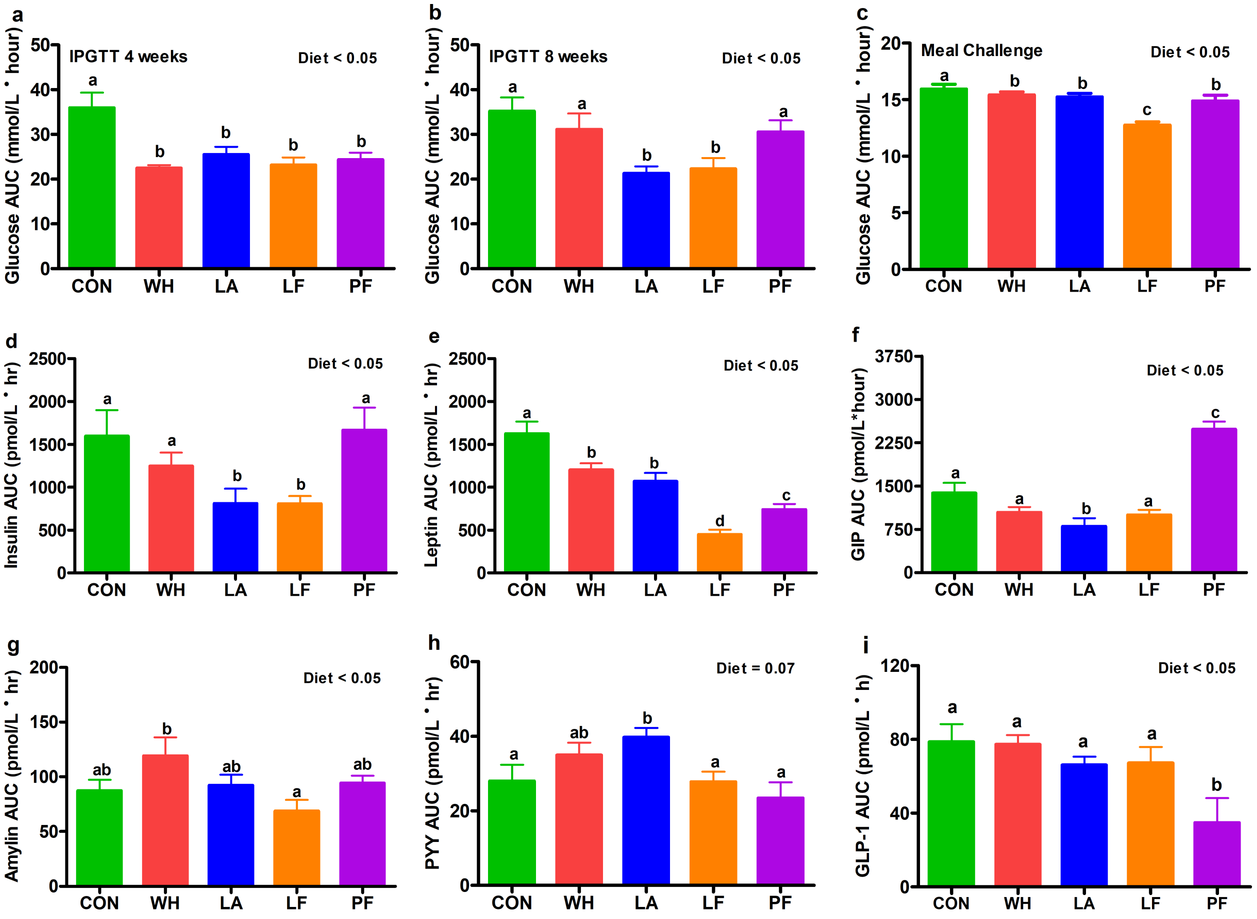

Supplementary Information

Supplementary Figure 3. Effects of whey, lactalbumin and lactoferrin on hepatic lipidosis. Hematoxylin-eosin stained (magnification 100X) and (**b**) Periodic Acid-Schiff stained (40X) liver sections from rats fed diets containing either control, whey protein isolate, α-lactalbumin, lactoferrin, or pair-fed to lactoferrin.

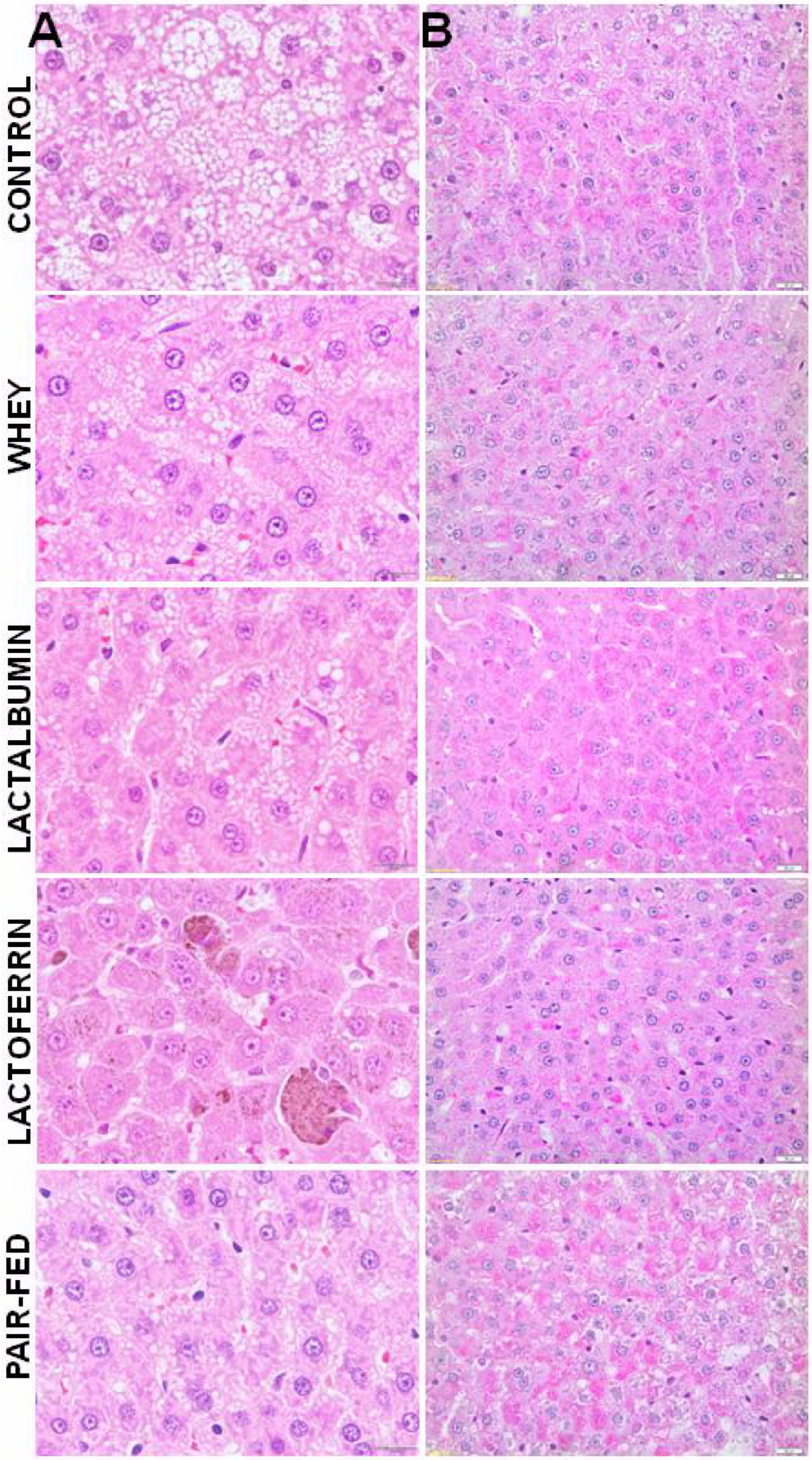

Supplementary Information

Supplementary Figure 4. Representative immunoblots for (**a**) Na$^+$-K$^+$-ATPase (**b**) Membrane-bound Insulin receptor beta chain (IRβ) and GLUT4 (**c**) Total IRβ and GLUT4 and (**d**) GAPDH in skeletal muscles from rats fed diets containing either normal protein (control) diet (CON), or high protein diets enriched with whey protein isolate (WH), α-lactalbumin (LA), lactoferrin (LF), or pair-fed to lactoferrin (PF). The samples derive from the same experiments, the gels and blots were processed in parallel, and the blots were re-probed for membrane bound and total IRβ and GLUT4. The animal numbers corresponding to each treatment (n = 6-8/treatment group) and molecular weight markers (RPN800E, GE Healthcare, Mississauga, ON, Canada) are also shown. Select bands from lanes 2-6 of each blot are cropped and presented in Figure 5.

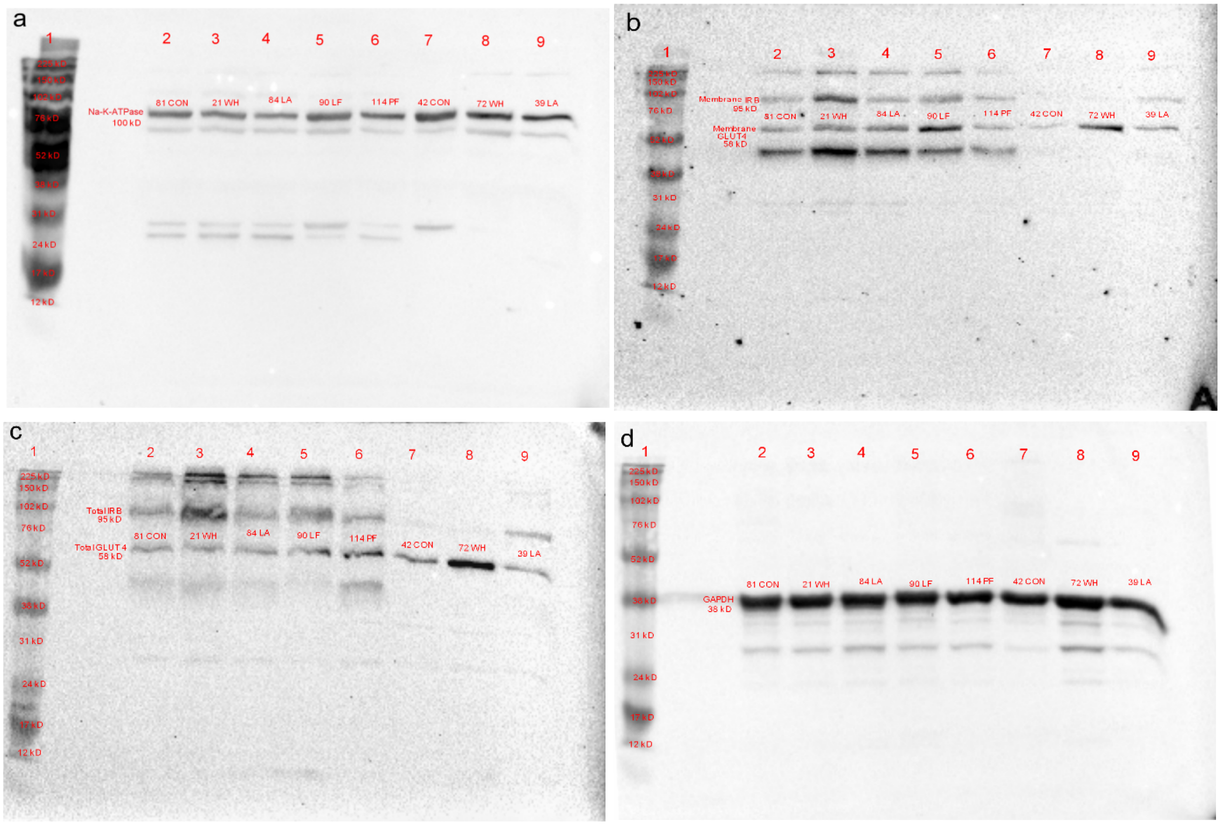

Supplementary Information

Supplementary Table 1. Tissue primers used in qRT-PCR.

**Gene***	**Primer Sequence (5'-3')**	**Location on template**	**Amplicon size (bp)**	**GeneBank Accession #**
**Slc2a2**	**F**: TTTGCAGTAGGCGGAATGG<br>**R**: GCCAACATGGCTTTGATCCTT	**F**: 337–355<br>**R**: 396–416	80	NM_012879
**Pfkl**	**F**: CTACCGTGGACCTGGAGAAGTTG<br>**R**: CCTGACAGCAGCATTCATACCTTG	**F**: 71-93<br>**R**: 171-148	101	NM_013190
**Pflm**	**F**: ATCGCCGTGTTGACCTCTGG<br>**R**: GCCTCCCTGATGTGCTCTCC	**F**: 87-106<br>**R**: 219-238	152	NM_031715
**Hk2**	**F:** GGTTTCAAAGCGGTCGAACT<br>**R:** TGGTCAACCTTCTGCACTTGG	**F:** 108-127<br>**R:** 267-247	160	NM_012735.2
**Gck**	**F:** GCAGAGCGGGTACTCACAAC<br>**R:** CTGCTCGACCCTCCACATGA	**F:** 191-210<br>**R:** 313-294	123	NM_001270849.1
**Gys1**	**F:** CCTCGGCTTACGGCATTTAC<br>**R:** GCAGGTCTGACAATCGCTCT	**F:** 1849-1868<br>**R:** 2001-1982	153	NM_001109615.1
**Gys2**	**F:** CCCTACCGCGTACGGTATTT<br>**R:** ATCTGAAAGCCTCTCGGTGC	**F:** 1861-1880<br>**R:** 2011-1992	151	NM_013089.2
**G6pd**	**F:** ATGACCCCACAGTACCCCAT<br>**R:** GGGTACTCCATCCCACCGTT	**F:** 1086-1105<br>**R:** 1174-1155	89	NM_017006.2
**Acaca**	**F**: ACCTCAACCACTACGGCATGA<br>**R**: AGGTGGTGTGAAGGCGTTGT	**F**: 3611–3631<br>**R**: 3665– 3684	74	NM_022193
**Fasn**	**F**: GGACATGGTCACAGACGATGAC<br>**R**: CGTCGAACTTGGACAGATCCTT	**F**: 171-192<br>**R**: 244-265	95	NM_017332
**Cpt1a**	**F**: GAGGCCTCCATGACAAGAATGT<br>**R**: GTGGACTCGCTAGTACAGGAA	**F**: 1807-1828<br>**R**: 1857-1877	71	NM_013200
**Pdha1**	**F**: CCTTTGGCTGGTTTTGGTTA<br>**R**: CACCAGTCATCAGCCTCAGA	**F**: 1080-1099<br>**R**: 1291-1272	212	NM_053551.1

**Ucp3**	**F**: AAAGGAACGGACCACTCCAG<br>**R**: CTTCACCACATCCGTGGGTT	**F:** 444-463<br>**R:** 542-523	99	NM_013167.2
**Ppargc1a**	**F**: GTGCAGCCAAGACTCTGTATGG<br>**R**: GTCCAGGTCATTCACATCAAGTTC	**F:** 56–77<br>**R:** 153–176	121	NM_031347
**Rps13**	**F**: GTTCTCTCTTTGCTTCCAGACC<br>**R**: CCCTCAGAATCACACCTATCTGG	**F:** 1-22<br>**R:** 197-175	197	NM_130432.2
**Actb**	**F**: GGATCAGCAAGCAGGAGTACGA<br>**R**: AACGCAGCTCAGTAACAGTCCG	**F:** 1148–1169<br>**R:** 1211–1232	85	NM_031144

***Slc2a2** - Glucose transporter – 2; **Pfkl** – Phosphofructokinase (Liver); **Pfkm** – Phosphofructokinase (Muscle); **Hk2** – Hexokinase; **Gck** – Glucokinase; **Gys1** – Glycogen Synthase 1; **Gys2** – Glycogen Synthase 2; **G6pd** – Glucose-6-Phosphate Dehydrogenase; **Acaca** – Acetyl-CoA Carboxylase 1; **Fasn** – Fatty Acid Synthase; **Cpt1a** – Carnitine palmitoyltransferase 1a; **Pdha1** – Pyruvate Dehydrogenase; **Ucp3** – Uncoupling Protein-3; **Ppargc1a** – peroxisome proliferator-activated receptor gamma co-activator 1α; **Rps13** – Ribosomal protein S13; **Actb** – actin, beta.

Supplementary Information

Supplementary Table 2. Host, dilution and source of primary and secondary antibodies used for immunoblotting.

Name*	Host	Dilution	Vendor and Catalogue#
**Anti-GLUT4**	Rabbit	1:1000	Millipore, Temiluca, CA; 07-1404
**Anti-IRβ**	Rabbit	1:200	Santa Cruz Biotech, Santa Cruz, CA; sc-711
**Anti-GAPDH**	Rabbit	1:200	Santa Cruz Biotech, Santa Cruz, CA; sc-25778
**Anti-Na^{+}/K^{+}-ATPase**	Rabbit	1:200	Santa Cruz Biotech, Santa Cruz, CA; sc-28800
**Anti-rabbit IgG**	Donkey	1:4000	GE Healthcare, Mississauga, ON; NA-934V

***GLUT4** – Glucose transporter-4; **IRβ** – Insulin receptor β-subunit; **GAPDH** - glyceraldehyde 3-phosphate dehydrogenase

Supplementary Information

Supplementary Table 3. Group or species-specific 16S targeted primers used for cecal microbial qPCR.

Bacteria	Primer Sequence (5'-3')	Amplicon size (bp)	Genomic DNA standard
Bacteroidetes/ Prevotella	**F**: GGTGTCGGCTTAAGTGCCAT<br>**R**: CGGACGTAAGGGCCGTGC	140	DSM 2079
Clostridium (Cluster I)	**F**: ATGCAAGTCGAGCGAGG<br>**R**: TATGCGGTATTAATCTCCCTTT	120	DSM 756
Clostridium (Cluster IV)	**F**: GCACAAGCAGTGGAGT<br>**R**: CTTCCTCCGTTTGTCAA	247	DSM 753
Clostridium (Cluster XI)	**F:** TTGAGCGATTTACTTCGGTAAAGA<br>**R:** CCATCCTGTACTGGCTCACCT	157	DSM 1296
Clostridium (Cluster XIV)	**F:** CGGTACCTGACTAAGAAGC<br>**R:** AGTTTCATTCTTGCGAACG	429	DSM 2950
Lactobacillus	**F:** AGCAGTAGGGAATCTTCCA<br>**R:** CACCGCTACACATGGAG	341	DSM 10533
Bifidobacteria	**F:** TCGCGTCCGGTGTGAAAG<br>**R:** CCACATCCAGCATCCAC	243	DSM 20083
Methanobrevibacter	**F**: CTCACCGTCAGAATCGTTCCAGTC<br>**R**: ACTTGAGATCGGGAGAGGTTAGAGG	111	DSM 861
Enterobactericeae	**F**: CATTGACGTTACCCGCAGAAGAAGC<br>**R**: CTCTACGAGACTCAAGCTTGC	190	DSM 30083
